# Insomnia symptoms and repressive coping in a sample of older Black and White women

**DOI:** 10.1186/1472-6874-7-1

**Published:** 2007-01-29

**Authors:** Girardin Jean-Louis, Carol Magai, Nathan S Consedine, Jessy Pierre-Louis, Ferdinand Zizi, Georges J Casimir, Louis Belzie

**Affiliations:** 1 Department of Psychiatry and Ophthalmology, SUNY Downstate Medical Center, NY, USA; 2 Brooklyn Research Foundation on Minority Health, KJMC, NY, USA; 3 Brooklyn Center for Health Disparities, SUNY Downstate Medical Center, NY, USA; 4 Department of Psychology, Long Island University, NY, USA; 5 Haitian American Psychiatric Association, NY, USA

## Abstract

**Background:**

This study examined whether ethnic differences in insomnia symptoms are mediated by differences in repressive coping styles.

**Methods:**

A total of 1274 women (average age = 59.36 ± 6.53 years) participated in the study; 28% were White and 72% were Black. Older women in Brooklyn, NY were recruited using a stratified, cluster-sampling technique. Trained staff conducted face-to-face interviews lasting 1.5 hours acquiring sociodemographic data, health characteristics, and risk factors. A sleep questionnaire was administered and individual repressive coping styles were assessed. Fisher's exact test and Spearman and Pearson analyses were used to analyze the data.

**Results:**

The rate of insomnia symptoms was greater among White women [74% vs. 46%; χ^2 ^= 87.67, p < 0.0001]. Black women scored higher on the repressive coping scale than did White women [Black = 37.52 ± 6.99, White = 29.78 ± 7.38, F_1,1272 _= 304.75, p < 0.0001]. We observed stronger correlations between repressive coping and insomnia symptoms for Black [r_s _= -0.43, p < 0.0001] than for White women [r_s _= -0.18, p < 0.0001]. Controlling for variation in repressive coping, the magnitude of the correlation between ethnicity and insomnia symptoms was substantially reduced. Multivariate adjustment for differences in sociodemographics, health risk factors, physical health, and health beliefs and attitudes had little effect on the relationships.

**Conclusion:**

Relationships between ethnicity and insomnia symptoms are jointly dependent on the degree of repressive coping, suggesting that Black women may be reporting fewer insomnia symptoms because of a greater ability to route negative emotions from consciousness. It may be that Blacks cope with sleep problems within a positive self-regulatory framework, which allows them to deal more effectively with sleep-interfering psychological processes to stressful life events and to curtail dysfunctional sleep-interpreting processes.

## Background

In the social science literature, ethnicity is often considered a proxy for socioeconomic constructs and sociocultural factors. In the context of sleep medicine, ethnicity might play a unique role in understanding insomnia symptoms. While many objective studies in the U.S. have suggested that individuals of African ancestry have characteristically worse sleep patterns [1,2], relative to those of European descent, the preponderance of evidence indicates lower rates of insomnia symptoms among the former. This is evident in two important epidemiologic studies: Duke's Established Populations for Epidemiologic Studies of the Elderly (≥ 65 years old) and the Cardiovascular Health Study of non-institutionalized Medicare enrollees. Specifically, in the Duke's studies 24% of older Blacks complained of sleep disturbances compared to 76% of older Whites [3]. This is consistent with data from the Cardiovascular Health Study, finding that 68% of Whites and 62% of Blacks reported nocturnal awakenings [4], although the contrast is les striking.

In a more recent study examining ethnic differences in the rate of insomnia symptoms from a community-based sample of older White (40%) and Black (60%) Americans, we found that ethnicity was the best predictor of sleep disturbances [5]. Factors entered in the regression model included sociodemographics, health risks, stress, social support, and mood. Regarding specific reports of insomnia symptoms: difficulty initiating sleep, difficulty maintaining sleep, and early morning awakenings, rates for White men and women were 41%, 75%, and 46%, respectively, whereas for their Black counterparts, rates were 14%, 37%, and 17% respectively. One explanation for this disparity was that reporting biases, commonly noted among older Blacks [6-8], might have influenced underreporting of insomnia symptoms [5]. Usually, reporting biases result from difficulties in understanding survey questions, poor recall, and social desirability. Conceivably, ethnicity alone might be inadequate as a proxy to explain differences in the rate of reported insomnia symptoms of older adults. This has prompted the need to explore the contribution of psychological factors affecting the likelihood of reporting insomnia symptoms.

Among older adults, several psychological factors (e.g., anxiety, depression, and worry) have been shown to affect the sleep process negatively [9]. It is well established that dysfunctional beliefs and attitudes about sleep play an important, mediating role in late-life insomnia [10-13]. In many cases, they not only predispose individuals to experiencing insomnia but also they can perpetuate it. Arguably, these factors may not of necessity bias the likelihood of reporting an insomnia symptom, although they are fundamental in the onset and maintenance of insomnia. Rather, the type of cognitive appraisal in which one engages while processing life stressors seems to affect the likelihood of experiencing sleep problems and/or biasing subjective report. There is evidence that negative appraisal of life stressors enhances the vulnerability to insomnia [14]. If in effect Blacks use more positive appraisal than do Whites [7,15,16], they may be less vulnerable to experiencing insomnia. Interestingly, appraisal among White individuals has the opposite effect; it often leads to increased psychophysiological distress [7].

In the present study, we tested the hypothesis that ethnic differences in the report of insomnia symptoms are mediated by variations in repressive coping styles. Repressive coping refers to individuals' ability to distance themselves psychologically from events discretely appraised as negative or situations that threaten their self-concept (see Methods). Lower rates of insomnia symptoms observed among Blacks might reflect a greater ability to regulate negative emotions about the sleep process. We also assessed the influence of health risks, medical morbidities, and beliefs and attitudes on sleep.

## Methods

Data presented in this paper were from a total of 1274 women participating in a community-based study in Brooklyn, NY. Of the volunteers, 28% were White, which consisted of U.S.-born White women and Eastern-European women from Russia, Ukraine, and Bellarus. The remaining 72% represented an aggregate of Blacks (i.e., U.S.-born African Americans and Caribbean Americans, including women born in Haiti, Barbados, Trinidad and Tobago, and Jamaica). The present analyses focus on differences between White and Black participants regarding sleep measures and repressive coping styles.

We used a stratified, cluster-sampling technique to gather representative data for the study. Accordingly, the Household Income and Race Summary Tape File 3A of the 1990 Census files were used to form census blocks representative of Brooklyn. Blocks were then stratified by ethnicity (Black and White) and by income (high, medium, and low). Once block groups were composed for each stratum, we randomly targeted samples for enrollment from each without replacement.

Trained, quality-controlled interviewers of the same ethnicity as the respondents administered several scales/questionnaires during face-to-face interviews conducted in the volunteer's home or a location of their choosing (usually a church or a senior center). Interviews lasted approximately 1.5 hours, and women completing the study received $25 for participating. The Internal Review Board at Long Island University approved the study.

Measures for the present analysis included demographic and health risk factors: age, ethnicity, education, income, BMI, smoking status, and alcohol consumption. Physical health was measured with the Comprehensive Assessment and Referral Evaluation (CARE). Repressive coping was assessed with the Index of Self-Regulation of emotion (ISE). Stress was measured by the Stress Index Scale, and volunteers provided information on health beliefs and attitudes.

The CARE has been widely used to assess physical health of older individuals in minority communities. It has shown good construct validity [17] as well as concurrent and predictive validity [18]; sub-scales included in our analyses were: somatic complaint, sleep disorder, leg problems, heart disease, respiratory disease, arthritis, vision problems, and hypertension (Cronbach α = 0.86; 0.85; 0.86; 0.83; 0.72; 0.91; 0.85; and 0.92, respectively).

Five questions comprise the sleep disorder subscale: "Do you depend on medicine to sleep?"; "Do you have difficulty falling asleep?"; "Do you wake up often during the night?"; "Do you wake up early and wake up feeling tired?"; and "Do you sleep during the day?". A subset of the volunteers (42%) also rated their sleep satisfaction on a scale from 1 (very satisfied) to 5 (very troubled), and estimated their habitual sleep duration, time spent in bed, time to bed, and final wake-up time. Insomnia symptom was defined as a report of either difficulty initiating sleep, difficulty maintaining sleep, or early morning awakening. No formal insomnia diagnosis could be formulated, as data on frequency, duration, or severity was not elicited.

The Index of Self-Regulation constitutes a modified version of Weinberger's conceptual model of repression [19]. According to Mendolia's research, the model stipulates that the interaction of individual differences in emotional responsiveness and situational threats to self-concept contributes to one's tendency to regulate emotional responsiveness. This model of repressive behavior posits that repressive copers are hypersensitive to both negative and positive emotional events, but they distance themselves from these events only when the situation threatens their self-concept [20-22]. Repressive coping refers to a person's belief that he or she is capable of conforming to rigid standards of self-control [23].

Mendolia's model represents an extension of conventional categorical measures of repression, which might not yield an accurate representation of observed variations in repressive coping [20]. This revised model accounts for the motivation and conditions in which repressors use a perceptual defense in response to negative and positive emotional events. In our analysis, ISE scores were derived following Mendolia's conceptualization, which amalgamates the defensive scale of the Social Desirability Scale (α = 0.73) [23] and the anxiety subscale of the State-Trait Anxiety Inventory (α = 0.75) [24]. Scores range from 0 to 52, with higher scores representing greater defensiveness/repressive coping. According to this classification scheme, individuals have a repressive coping style when they are highly defensive (e.g., high score on the social desirability) scale, but also low in trait anxiety (e.g., low score on the manifest anxiety scale). Details on the derivation of ISE scores as used in our analysis have been reported elsewhere [25].

The Stress Index Scale used initially by the National Survey of Black Americans was administered to our participants [26]. Respondents rated on a 4-point scale the degree to which a set of items provoked stress in the past month or two. These stress-induced life events were health, money, job, problems with family or marriage, problems with people outside the family, children, crime, police, love life, and racial conflict. Scores ranged from 0 to 29, and higher scores denoted greater stress levels (α = 0.81). Additionally, for the purpose of our analyses we selected five questions from the 'Health Practices' and 'Beliefs and Attitudes Toward Health' questionnaires. These included three items assessing help-seeking behavior: 1) seeking help from a religious healer, 2) seeking help from a spiritualist or a neighborhood healer, and 3) regular annual physical exams. The other two assessed religious faith as a coping strategy to deal with life challenges: 2) praying is the best way to cope with health problems and 2) God will take care of me when ill.

### Statistical analysis

Frequency and measures of central tendency were used to describe the sample. Variables were examined for normality and tested for collinearity, and those that were non-gaussian were transformed using appropriate statistical techniques. Analysis of variance was used to assess ethnic differences on continuous variables. Fisher's Exact test was employed to assess effects of ethnicity on insomnia symptoms, health measures, help-seeking behavior, and religious faith.

Spearman correlations were used to explore relationships between ethnicity and sleep measures and ISE scores and between ISE scores and sleep measures. Since ethnicity and ISE were correlated, we performed partial correlations to assess associations of ethnicity with sleep measures, controlling for variations in ISE scores. In separate partial correlation analyses, we explored whether differences in demographic factors (i.e., age, education, income, and marital status) or health factors (i.e., somatic complaint, heart disease, respiratory disease, arthritis, leg problem, vision problem, and hypertension) would have any mediating effects on associations of ethnicity with sleep measures. We also examined whether variations in help-seeking behavior and religious faith would influence these associations. Furthermore, relationships of ethnicity and ISE scores to insomnia symptoms were also examined using logistic regression analysis.

Since we examined relationships between many variables, we assessed a priori what significance criterion could be accepted using an observed probabilities plot (*p*-plot) [27]. The *p *-plot indicated that correlations with a probability less than 0.04 could be regarded as unlikely to be due to chance. Thus, acceptance of probabilities ≤0.01 in the present analyses represents a conservative criterion, thus limiting the likelihood of a Type I error. This is consistent with application of a Bonferroni adjustment to the largest correlation matrix (nine variables) in our analyses, which indicated that probabilities ≤0.01 would be acceptable.

## Results

In Table 1, we compare the demographic and health risk characteristics of the sample based on ethnicity. Significant ethnic differences in income, percent of women with a high school degree, percent married, and percent of social drinking were found, but no significant ethnic effect on the rate of current smoking was found. Stress levels were comparable for Blacks and Whites [Black = 9.32 ± 6.61 and White = 9.80 ± 5.66, F_1,1273 _= 11.48, NS]; thus, stress was not considered in partial correlations. As seen in Table 2, White participants reported significantly greater rates of somatic complaints, respiratory problems, arthritis, heart diseases, and leg problems, whereas Blacks reported significantly greater rates of hypertension and vision problems.

**Table 1:** Demographic and Health Risk Characteristics of Participants

**Variable**	**Blacks (72%)**	**White (28%)**	**F/χ**^**2**^
**Mean Age**	59 ± 7	60 ± 6	4.04
**Mean Household Income, K**	29 ± 21	38 ± 29	31.83**
**Mean Body Mass Index**	30 ± 6	28 ± 6	18.31**
**% Married**	30	51	46.16**
**% High School Degree**	76	99	93.44**
**% Current Smoking**	12	11	0.24
**% Social Drinking**	18	34	33.25 **

**indicates significant differences using ANOVA or Fisher's Exact test at alpha = 0.01

**Table 2:** Comparison of Reported Health Problems by Ethnicity

**Variable**	**Black (72%)**	**White (28%)**	**χ**^**2**^
**Respiratory Disease (%)**	29	52	60**
**Hypertension (%)**	57	41	26**
**Heart Disease (%)**	41	55	20**
**Arthritis (%)**	67	73	4**
**Leg Problem (%)**	52	61	8**
**Vision Problem (%)**	54	28	67**
**Somatic Complaint (%)**	60	81	52**

**indicates significant differences using Fisher's Exact test at alpha = 0.01

We found no significant ethnic differences in help-seeking behavior (see Table 3). Despite the fact that more Blacks believe that a religious or spiritual healer can treat illnesses, they did not disproportionately seek their help. A significantly higher percentage of Blacks believed that prayer is the best way to cope with health problems and that God takes care of them when ill. Although more Blacks believed that herbal medicines have healing values, few of those sharing these beliefs actually used herbs to treat mild health conditions [14%] and even fewer for serious conditions [1.5%]. Among Whites with similar beliefs, 39% used herbs for mild conditions and 1.9% for serious conditions.

**Table 3:** Comparison of Help-Seeking Behavior and Religious Faith by Ethnicity

**Variable**	**Black (72%)**	**White (28%)**	**χ**^**2**^
**Annual Physical Exam (%)**	87	85	0.94
**Help from a Religious Healer (%)**	5	5	0.06
**Help from a Spiritualist (%)**	2	3	0.54
**Healers Treat Health Problems (%)**	35	18	38**
**Herbs Have Healing Values (%)**	58	44	20**
**Prayer Helps with Health Problems (%)**	85	31	342**
**God Takes Care of Me When Ill (%)**	77	34	208**

**indicates significant differences using Fisher's Exact tests at alpha = 0.01.

Overall, the percentage of White women reporting insomnia symptoms was greater than that of Blacks [74% vs. 46%; χ^2 ^= 87.67, p < 0.0001; r_s _= 0.26, p < 0.001]. Rates for each sleep-related complaint are compared in Table 4, showing significantly greater rates for Whites with regard to difficulty initiating sleep, difficulty maintaining sleep, early morning awakening, and use of sleep medicine, except for daytime sleep, which was more prevalent among Blacks. Moreover, Black ethnicity correlated with greater sleep satisfaction [r_s _= 0.20, p < 0.001], and earlier rise time [r_s _= 0.18, p < 0.001] and bedtime [r_s _= 0.17, p < 0.001]. Trends suggested longer sleep duration and better sleep quality for Blacks as well [r_s _= -0.10, p = 0.04; r_s _= -0.11, p = 0.02, respectively].

**Table 4:** Comparison of Insomnia-Related Symptoms by Ethnicity

**Variable**	**Black (72%)**	**White (28%)**	**χ**^**2**^
**Difficulty Initiating Sleep (%)**	16	42	86**
**Difficulty Maintaining Sleep (%)**	40	64	60**
**Early Morning Awakening (%)**	27	53	81**
**Daytime Sleep (%)**	9	4	9*
**Sleep Medicine (%)**	4	19	83**

**indicates significant differences using Fisher's Exact tests at alpha = 0.01; * at alpha = 0.05.

Generally, women with greater scores on the repressive coping scale (ISE) reported fewer insomnia symptoms [r_s _= -0.44, p < 0.001], greater sleep satisfaction [r_s _= -0.28, p < 0.001], slept longer [r_s _= 0.20, p < 0.001], and had better sleep quality [r_s _= 0.34, p < 0.001]; bedtime and rise time were relatively unaffected by variations in repressive coping. Since Black women showed greater ISE scores than White women [Black = 37.52 ± 6.99 and White = 29.78 ± 7.38, F_1,1272 _= 304.75, p < 0.0001], we examined relationships of ISE with insomnia within each ethnic group. Black women characterized by greater ISE scores reported lower rates of insomnia [r_s _= -0.43, p < 0.0001]. Within the White ethnicity, women with greater ISE scores also reported lower rates of insomnia [r_s _= -0.18, p < 0.0001]. Notably, the magnitude of the correlation coefficient was smaller, but this was not confounded by larger cell size for the Black group.

Since both ISE and ethnicity were associated with sleep measures, and that Black women tended to have greater ISE scores, we assessed whether ethnicity would remain a significant correlate of sleep measures controlling for variations in ISE scores. Partial correlation analyses showed that the magnitude of the relationship between ethnicity and insomnia, sleep satisfaction, rise time, and bedtime was substantially reduced [r_p _= 0.09, p < 0.01; r_p _= 0.10, p < 0.01; r_p _= 0.14, p < 0.01; r_p _= 0.08, p < 0.01, respectively]. Correlations with sleep duration and sleep quality were too weak for any meaningful interpretation; thus, they were not included in partial correlations.

Controlling for effects of age, income, education, marital status, and BMI did not result in significantly lower correlations between ethnicity and insomnia, sleep satisfaction, and rise time [r_p _= 0.22, p < 0.01; r_p _= 0.17, p < 0.01; r_p _= 0.17, p < 0.01, respectively], except for bedtime [r_p _= 0.07, NS]. Similarly, with control for variations in help-seeking behavior and religious faith, we did not find substantial changes in the correlations [r_p _= 0.20, p < 0.01; r_p _= 0.17, p < 0.01; r_p _= 0.14, p < 0.01, respectively], except for bedtime [r_p _= 0.06 NS]. With multivariate adjustment for differences in health characteristics, the correlations were largely unaffected: [r_p _= 0.19, p < 0.01; r_p _= 0.13, p < 0.01; r_p _= 0.16, p < 0.01, respectively]; for bedtime, it was [r_p _= 0.05, NS].

Given the ethnic disparity in medical morbidities (see Table 2), we assessed independent associations of ethnicity and ISE scores with insomnia symptoms using logistic regression analysis with insomnia symptoms as a binary variable. These analyses indicated that being White [OR = 1.79; CI: 1.32–2.41] and a low regulator (low ISE scores) [OR = 5.30; CI: 4.09–6.87] were significant independent predictors of the likelihood of reporting insomnia symptoms. With multivariate adjustment for medical factors, adjusted odds ratios for ethnicity and ISE were 2.01 (CI: 1.39 – 2.92) and 2.84 (CI: 2.09 – 2.85), respectively. Adjusted odds ratios for each of the medical morbidities are indicated in Table 5; somatic complaint was the most important contributor.

**Table 5:** Associations Between Medical Factors and Insomnia Symptoms

**Variable**	**Wald**	**OR**	**CI**
**Arthritis**	17.91**	2.10	1.49 – 2.97
**Hypertension**	0.17	1.07	0.77 – 1.50
**Heart Disease**	2.51	1.33	0.94 – 1.88
**Vision Problem**	3.00	1.32	0.96 – 1.82
**Respiratory Disease**	28.83**	2.35	1.72 – 3.21
**Leg Problem**	19.66**	2.05	1.49 – 2.82
**Somatic Complaint**	34.68**	2.81	1.99 – 3.96

Adjusted odds ratios (OR) derived from regression of the insomnia measure on medical factors. ** p < 0.01

## Discussion

### Ethnic differences in rates of insomnia symptoms

The main objective of our research was to ascertain which factors are associated with sleep problems among older adults living in Brooklyn communities. The first observation in the present study was that Black women reported significantly fewer insomnia symptoms than did White women. This was also true in our previous study of Brooklyn residents, despite the age disparity in the sample [5]. One implication of this finding is that ethnic effects on rates of insomnia symptoms are also observable among younger women. There is no reason to suspect that similar effects would not be found among younger men as well. This finding is consistent with available epidemiologic evidence that Blacks generally report fewer insomnia symptoms relative to age-matched Whites [3-5]. In all, the proportion of White women in Brooklyn, NY reporting insomnia symptoms was 62% greater than for Black women.

Other related findings were that Black women seemed to retire and got up earlier than did Whites and that Blacks might have experienced longer and more satisfying sleep than their White counterparts. Trends regarding longer sleep time for Blacks are in line with data from the National Health and Nutrition Examination Survey, showing that more Blacks (11%) than Whites (8%) reported sleeping more than 8 hours [28], the recommended sleep time by the National Sleep Foundation [29,30]. About a decade earlier, the National Health Interview Survey estimated that sleep length greater than 8 hours was also greater among Blacks (18%) relative to Whites (11%) [31]. On balance, we should also consider evidence suggesting that sleeping less than 6 hrs might be more prevalent among Blacks [31]; thus sleep time might be more variable among Blacks. Definitive conclusions regarding longer sleep time among Blacks await objective, population-based studies.

While the amount of sleep experienced by the U.S. adult population decreases steadily [32,33], sleep complaints seem to have increased commensurately [34], causing public health advocates to be concerned about the likelihood that different segments of the population might be at greater risks for adverse effects of sleep restriction [35-38]. A cursory view of the available epidemiologic evidence within the context of ethnicity would have suggested that Blacks are at lower risks compared to Whites, as the former report fewer insomnia symptoms and being generally more satisfied with their sleep. However, considering clinical evidence that White patients often sleep longer than do Blacks with the same sleep disorder diagnosis [1,39,40], it is unclear whether Blacks are in fact at lower risks for adverse effects of sleep loss.

Absent empirical verification of those findings then, we cannot affirm with certitude why Blacks reported fewer insomnia symptoms. Initially, we had suspected that higher rates of insomnia symptoms among White women could be attributed to their report of more medical comorbidities as reflected by higher rates of arthritis, respiratory disease, and heart disease; these are often associated with sleep disturbances. However, based on regression analysis ethnic effects persisted even after multivariate adjustment for ethnic disparities in physical health. It does not appear that differences in reported sleep or health complaints are explainable on the basis of differing socioeconomic or immigration status [41-43]. Analysis of the previous Brooklyn data revealed that such factors were not significant independent predictors of sleep disturbance [5]. Differences could not be explained by ethnic variation in help-seeking behavior, as Blacks were as likely as Whites to receive a yearly physical exam. We observed important differences in religious and cultural beliefs [44], but they did not have independent effects on sleep profiles. Although women in both groups believed that prayer is the best way to cope with health problems, belief in God did not influence rates of insomnia symptoms [45].

### Ethnicity and insomnia symptoms: influence of repressive coping

The second main finding of the study is that the relationship of ethnicity to insomnia and sleep satisfaction is jointly dependent on the degree of repressive coping. With control for effects of repressive coping, the magnitude of the relationship of ethnicity to insomnia and sleep satisfaction diminished, indicating that repressive coping was indeed a mediating factor. Such was not the case when we controlled for effects of socidemographics, medical factors, health risk characteristics, or help-seeking behavior. This suggests that Black women reported fewer insomnia symptoms because of a greater ability to regulate their emotions. It may be that Blacks cope with sleep problems within a positive self-regulatory framework, which allows them to deal more effectively with sleep-interfering psychological processes to stressful life events and to curtail dysfunctional sleep-interpreting processes [12,14]. If indeed Blacks are better appraisers of daily stressors negatively affecting the sleep process, this attribute would reduce their vulnerability to insomnia particularly where anxiety constitutes the dominant feature [14]. It is of interest to determine whether self-reported and physiologically monitored sleep patterns differ greatly among individuals showing divergent repressive coping styles.

The explanation for the lower rates of insomnia symptoms among Blacks formulated in our previous report pointed to appraisal research [5], which shows that Blacks use more positive reappraisal than do Whites [7,8,16]. In light of new data, we revise our previous explanation using repression theories. The current explanation does not negate the one previously articulated; it clarifies it. The finding that Blacks report fewer insomnia symptoms does not reflect of necessity a response bias, as might be motivated by greater social desirability, although repressors are inclined to behave in socially desirable ways [46]. It does not appear that our method of gathering data offered the motivational and situational antecedents to elicit such behavior. Trained staff of the same ethnicity as the respondents conducted interviews soliciting non-threatening information in a place of their choice. Besides this consideration, one imagines that the resulting incongruence between underreporting of sleep problems and the actual sleep experience, if deemed unsatisfactory, would probably be itself a precipitating factor in the onset of insomnia. Generally, Blacks may not be underreporting insomnia symptoms at all. Rather, the regulatory style they employ as they approach the sleep experience may actually serve to offset the adverse effects of negative affect on sleep.

There may be two mechanisms by which the sleep experience of older Blacks differs from that of older Whites. It is likely that life stressors, which ordinarily induce negative cognitive-affective dispositions, do not readily influence the sleep process of highly regulated Blacks. In this regard, the underlying motivation for repressive coping is to protect the sleep process by not permitting access to the memory of negative emotional events, potentially impinging on one's ability to initiate and/or maintain sleep. This would be in line with the view that late-life insomnia is perpetuated by negative attitudes and beliefs about sleep, which can be successfully addressed with cognitive behavior therapy. As adapted by sleep clinicians, the use of this therapeutic modality facilitates reduction of dysfunctional beliefs (or self-perceived stress), which can obviate insomnia symptoms even among individuals with severe insomnia [10,11].

It is equally likely that Black repressors are more adroit at distancing themselves psychologically from a sleep experience discretely appraised as negative [20]. Regardless of the type of sleep experienced, it would be deemed satisfying, thus forestalling any triggers of conditioned insomnia resulting from a negative emotional reaction to poor sleep. This idea is consistent with previous findings that some self-described normal sleepers may endure severe sleeping difficulty with no corresponding reports of insomnia symptoms [47,48]. Repressive coping in this context would entail a greater dissociation from the somatic reaction to poor sleep and the personal distress with which it is associated [19,22]. It was interesting that not only did Black women show higher self regulation, they also reported fewer somatic complaints, supporting a previous report of attenuated somatic symptomatology among repressors [49]. If indeed, Black repressors are *poor encoders *of negative experiences, as would be predicted by thought-suppression experimental paradigms [20,46,50], the failure to report insomnia symptoms may in fact imply poor recall ability due to inadequate depth of processing or retrieval inhibition. One wonders whether repressors with an insomnia diagnosis could accurately assess their sleep profile immediately upon experimental awakening permitting no opportunity to maintain congruence with previous reports of sleep profile.

In sum, each of the proposed mechanisms employs inhibitory strategies, one preempting the devastating effects of negative cognitive-affective dispositions on sleep, and the other disallowing any perpetuating effects of a negatively appraised sleep experience. Whatever the mechanism at work, their modus operandi is to reduce the likelihood that sleep problems would predominate the awareness of repressive copers.

Whereas Blacks with insomnia symptoms may benefit from this unique ability to cope with challenges posed by sleep disturbances, this may be maladaptive among those suffering from sleep apnea or other medical conditions causing insomnia. There are data suggesting that the repressive coping style, consisting of a constant presentation of a highly positive and optimistic self-image, may be psychologically maladaptive [51]. Repressive individuals reporting low level of anxiety have been found to exhibit high level of anxiety based on physiological tests, and some may be unusually sensitive to anxiety-provoking information [51,52]. Perhaps, the propensity not to address sleep problems might explain in part why sleep apnea is a public health problem in Black communities [2]. One population-based study, comparing community-dwelling older Blacks and Whites, found that Blacks were experiencing severe sleep apnea with a relative risk twofold as great (relative risk = 2.13) as that of their White counterparts [1]. Yet, data we collected at a sleep clinic in Brooklyn suggest that only 35% of Blacks are likely to comply with a doctor's recommendation to undergo a polysomnographic evaluation. This is quite alarming since 91% of Black patients in that clinic who consented to be evaluated received a diagnosis of sleep apnea [53]. It is estimated that more than half of Blacks with sleep apnea may be unaware of it.

## Conclusion

Plausibly, gradual sleep loss along with commensurate increases in insomnia symptoms [34,54] affects Blacks and Whites proportionately. Which group is at greater risks for adverse events is yet undetermined. Results of our study lead to the realization that, on the one hand, the ability of older Blacks to cope more effectively with insomnia challenges, if proven, would constitute an important asset. On the other hand, the failure to recognize sleep disturbances when accompanied by medical and/or psychiatric comorbidities represents a liability for Blacks.

This liability is manifest when we consider the consequences of untreated sleep apnea, which might include increased odds of involvement in a motor vehicle accident and vulnerability to hypertension, cardiovascular diseases, and type 2 diabetes mellitus, or early mortality [55-65]. Since individuals belonging to the Black ethnicity are at greater risks for sleep apnea, they would benefit from increased efforts to improve awareness of the importance of early detection of this condition. Whereas there is no direct evidence to encourage Blacks to sleep longer than the current population mode [33], it seems a prudent practice that they avoid acute sleep loss as they may be at greater risks for adverse events.

## Competing interests

The author(s) declare that they have no competing interests.

## Authors' contributions

**GJL **formulated hypotheses of the study, performed data analysis, and drafted the manuscript.

**CM **designed the main study and assisted in drafting of the manuscript.

**NSC **participated in interpretation of results and assisted in the drafting of the manuscript.

**JPL **participated in training interviewers and coordinated data acquisition, coding and cleaning. She also took part in reviewing the manuscript.

**FZ **assisted in interpretation of results and reviewed the manuscript.

**GJC **participated in interpretation of results and reviewed the manuscript.

**LB **participated in interpretation of results and reviewed the manuscript.

All authors read and approved the final manuscript.

## Pre-publication history

The pre-publication history for this paper can be accessed here:


